# Knowledge of Postgraduate Medical Trainees Regarding Epidemiology

**DOI:** 10.7759/cureus.2171

**Published:** 2018-02-08

**Authors:** Shoaib Shafi, Tayyaba Faisal, Sajida Naseem, Sajida Javed, Haider Ghazanfar

**Affiliations:** 1 Medicine, College of Physician and Surgeons Pakistan; 2 Research Evaluation Unit, Regional Center Islamabad, College of Physician and Surgeons Pakistan; 3 Department of Community and Family Medicine, Shifa International Hospital, Islamabad, Pakistan; 4 Publications, Army Medical College, Rawalpindi; 5 Internal Medicine, Shifa College of Medicine, Islamabad, Pakistan

**Keywords:** epidemiology, post graduate trainee, evidence based medicine, medical education

## Abstract

Introduction

An understanding of epidemiology is fundamental for designing research and understanding evidence-based medicine (EBM). The purpose of our study was to determine the knowledge of epidemiology among postgraduate medical trainees.

Methods

We carried out a cross-sectional study from January 2017 to June 2017 at the Regional Centre Islamabad, College of Physicians and Surgeons Pakistan. About 204 postgraduate medical trainee were enrolled in the study after taking written consent. All of the participants were working as a postgraduate medical trainee in different hospitals of Rawalpindi and Islamabad. A self-designed structured questionnaire containing 20 multiple choice questions regarding knowledge of epidemiology was given to all the participants. Knowledge of epidemiology was measured and compared with gender, hospital, and previous history of research. Statistical Package for the Social Sciences (SPSS), version 21.0 (IBM, Armonk, NY) was used for data analysis.

Results

Out of 204 participants, 46.1% participants were males while 53.9% were females. The mean age of the participant was 28.5 ± 2.5. Majority fo the trainees (33.8%) were training in an Internal Medicine residency program. Most of the trainees (76%) had average knowledge of epidemiology while 18.6% of the trainees had good knowledge. Only 17.6% had some experience of research projects in the past, but this was not related to knowledge of epidemiology (p > 0.05).

Conclusion

Only 18.6% of the postgraduate trainees had good knowledge regarding epidemiology and 76% had average knowledge. This knowledge did not relate to their gender, hospital settings, and previous experience of conducting research proposals and articles.

## Introduction

Epidemiology is absolutely necessary for the understanding of disease etiology and prevention of disease by specific measures such as immunization and screening. In the era of evidence-based medicine (EBM), we all depend on the knowledge gained through epidemiological studies [1]. Majority of the doctors (95.5%) believed that EBM is important for the practice of medicine [2]. Medical education is mostly aimed at preparing doctors to respond to the needs and requirements of sick individuals [3]. The main source of information for EBM is research. These research studies help in better management of the patients [4]. In a study done by Daher AM et al. on 138 students, 80.7% of the students believed that the subject of epidemiology plays a fundamental role in understanding and solving basic health issues [5].

Study of epidemiology teaches us how to design and carry out research studies. There are multiple study designs available to carry out research. According to a study, the most common study design was the cross-sectional study (40%) followed by the retrospective study (18%) and randomized controlled trial (8%) [6]. Studies have shown that appropriately 30% to 90% of the studies have flaws in their designs and statistical analysis [4-5,7].

Students who are more involved in research during medical school are more likely to actively participate in postgraduate research activities. Prior undergraduate knowledge and experience of research can increase the understanding of research studies [8]. According to Khalid M et al., 91% of the students believed that research methodology should be part of the medical school curriculum. According to the participants in this study, deficiency in training courses (88.8%), lack of supervisors (84.7%), lack of time (72.3%), and no funding support (54.1%) were the main obstacles in doing research during medical schools [9]. Research experience during medical school can help the students by improving their abilities to do a proper literature review and also help them in doing a critical appraisal of articles which can lead the student to establish a good career and become a better resident and fellow [10]. A study done in Pakistan showed that out of 126 students from the third and fourth year; 76% of the student believed that epidemiology should be taught in the first two years of medical school [11].

It is important to evaluate the knowledge of epidemiology in postgraduate medical trainees as this is the time when we can train them and improve their knowledge and skills regarding epidemiology. Trainees who do not have sufficient knowledge regarding epidemiology are not able to conduct research activities and practice EBM [12]. The objective of this study was to determine the knowledge of epidemiology among postgraduate medical trainees. To the best of our knowledge, very few studies are available in our region regarding the evaluation of knowledge of epidemiology.

## Materials and methods

We carried out a cross-sectional study from January 2017 to June 2017 among postgraduate medical trainees working in hospitals of Rawalpindi and Islamabad. The participants were selected through the consecutive non-probability sampling technique. A total of 204 participants were included. The sample size of 204 was calculated using the World Health Organization (WHO) calculator keeping confidence level at 95%, anticipated population proportion for knowledge of residents regarding epidemiology methods at 45.6% [4] and absolute precision at 7%. Informed consent was obtained from all the participants. A self-designed structured questionnaire containing 20 multiple choice questions regarding the knowledge of epidemiology was given to all the participants. Cronbach's Alpha value for the questionnaire was found to be 0.87.

The first part of the questionnaire compromised of six questions which were based on the demographic information of the participants. The first part covered questions regarding age, gender, hospital setting, the name of the medical school from where they graduated, year of training, and previous experience of conduction research. The second part of the questionnaire compromised of 20 multiple choice questions regarding knowledge about epidemiology. Questions with missing answer were considered incorrect. The total score of the second part was categorized into good, average, and poor. A score from zero to seven was considered as poor while a score from eight to fourteen and fifteen to twenty was considered as average and good, respectively. Each participant was given 20 min to answer the questions. The data collection was done under the supervision of the researchers.

The data of the study was analyzed using the Statistical Package for the Social Sciences (SPSS), version 21.0 (IBM, Armonk, NY). Quantitative variables like age were measured as the mean and standard deviation. Descriptive statistics were measured for qualitative variables. Chi-square test was applied to measure the association of knowledge with age, gender, hospital setting they were working in, the medical school from where they graduated, year of training and previous experience of conducting research. P- value < 0.05 was taken as significant.

## Results

About 204 respondents who had given informed consent were included in the study. The response rate was 100. Out of 204 students; 94 (46.1%) were males while 110 (53.9%) were females. The mean age of the participants was 28.5 ±2.5 years. About 144 (70.6%) of the participants were doing the residency at public sector hospital while 60 (29.4%) were doing the residency at private sector hospitals. Majority of the participants (total of 69; 33.8%) were in their residency in internal medicine while 56 (27.5%) participants were doing the residency in Surgery, 25 (12.3%) in Gynecology and Obstetrics, 18 (8.8%) in Pediatrics, 13 (6.4%) in Opthalmology, 11 (5.4%) in Dentistry, seven (3.4%) in Pathology and five (2.5%) in Otorhinolaryngology. Out of 204 trainees, 78 (38.2%) and 111 (54.4%) were in the first and second year of training while 13 (6.4%) and 2 (1%) were in the third and fourth year of training. About 157 (77%) had graduated from their medical school one to five years ago while 42 (20.5%) and five of them (2.5%) had graduated from their medical school six to ten years ago and more than 10 years ago respectively. Majority (121; 59.3%) of the participants had done their graduation from government colleges. Only 36 (17.6%) of the postgraduate trainees had some experience of doing research projects.

**Figure 1:**
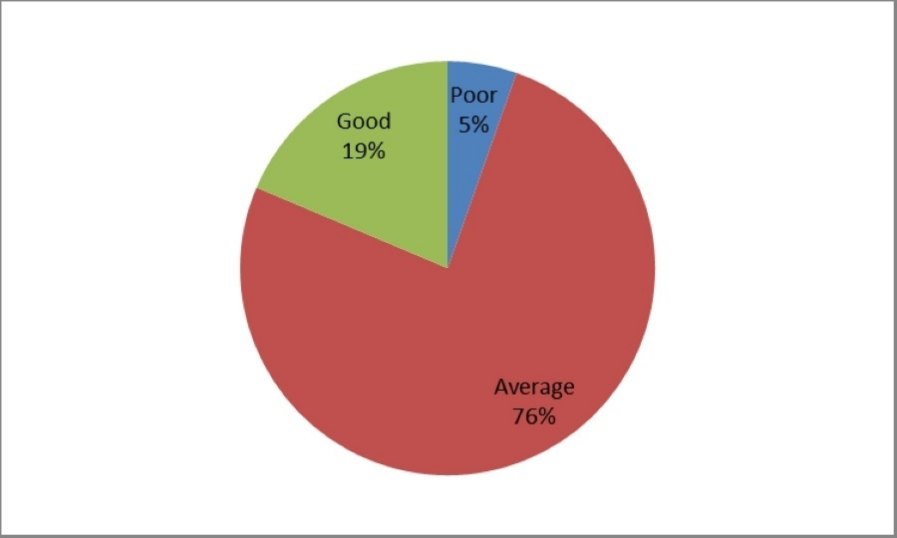
Knowledge of epidemiology among postgraduate medical trainees

Majority of the participants (155; 76%) had average knowledge of epidemiology while 38 (18.6%) had good and 11 (5.4%) poor knowledge of epidemiology, respectively. This has been presented in Figure 1.

**Figure 2:**
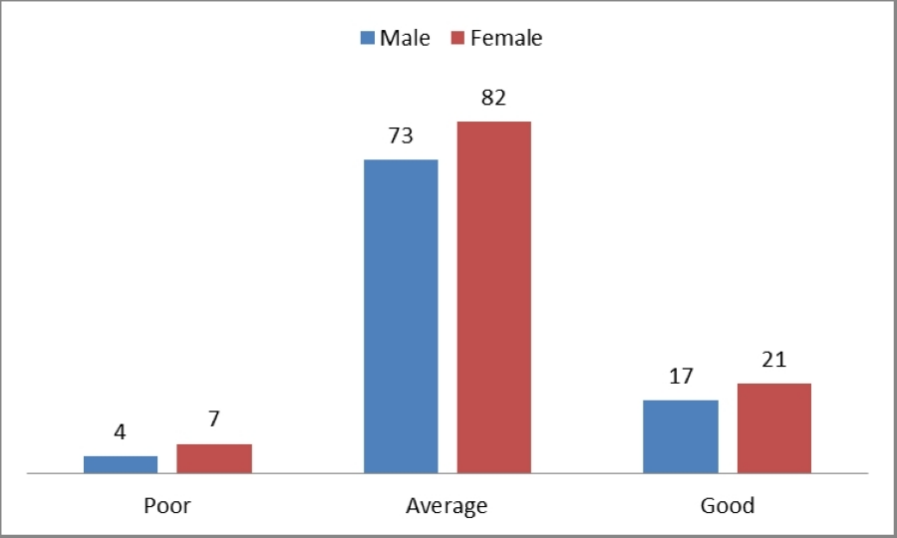
Gender-wise distribution of the knowledge of epidemiology among postgraduate medical trainees

**Figure 3:**
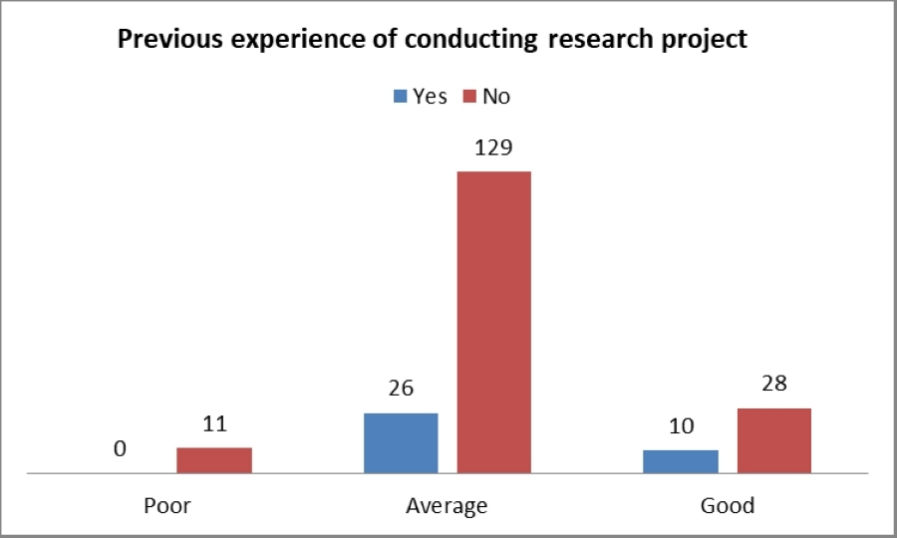
Knowledge of epidemiology among postgraduate medical trainees with respect to previous experience in research

None of the participants with a prior experience of research project had poor knowledge. There was no significant association ( p-value > 0.05) between knowledge of epidemiology with respect to gender, type of hospital they were being trained, and previous experience of conducting any research project. This has been presented in Figure 2 and Figure 3.

## Discussion

Our main objective of this research was to measure the knowledge of the postgraduate medical trainees regarding epidemiology. The response rate in our study was 100% as compared to other studies in which it was 76% [8], 77% [12] and 84.2% [4]. Among 204 students in our research, 46.1% were male and 53.9% were female, showing more females participants as compared to males participants which is different as compared to the study done by Segal S [8] in which there were more male participants. Another study done showed a predominance of female participants (56%) [12]. The mean age of the participants in this study was 27 ± 7 years (24-45 years). In our study, the mean age of the participants is 28.5 ±2.5 years.

A study done by Zambudio AR et al. showed a similar portion of participants training in different specialties as compared to our study. According to the study by Zambudio AR, 47% of the participants were training in internal medicine while 22% were training in surgery and 31% were training in central services; while specialty was not specified in the remaining (0.6%) of the participants. Internal medicine was also the most common training specialty in another study conducted by Novack L et al. [4]. In terms of the year of residency, Novack L et al. showed 22% were in the first year, 25% in the second year, 26% in the third, 20% in the fourth, and 5% in the fifth year [4]. In our study, most of the trainees were in the first and second year.

There was no difference in the knowledge between male and female trainees which was rated as good, average and poor in males (18.08% 77.6%, 4.2 %%) and (19.09%, 74.5%, 6.3%) females. This was comparable with the study conducted by Segal S [8]. According to a study done in India, 58% of the trainees had fairly good knowledge about epidemiology and research; and 76% of the participants agreed that teaching about research should be part of the training. This study also suggested that improvement in the medical education system is mandatory to flourish research culture [13]. This result was consistent with a study done in Pakistan where 70.9% of the participants wanted to engage in research activities but could not pursue it because of lack of training [14].

A study done at the Aga Khan Hospital in Pakistan concluded that out of 187 participants only 26.9% had research experience during their residency program and limited time was the most commonly cited reason for lack of research activities [15]. In the same study, it was the concluded that residents had inadequate knowledge of research; the residents were of the opinion that the curriculum should be modified in order to incorporate meaningful research experience [15]. A study done in India concluded that the residents had inadequate knowledge regarding epidemiology but had a positive attitude towards learning [16]. In contrary, a study done in Iran showed that 77.8% of the residents had favorable knowledge but had a negative attitude towards further learning [17]. 

According to other studies, having a higher level of knowledge about epidemiology was associated with shorter elapsed time since graduation, a higher number of publications, and self-reported reading. A study concluded that 53.7% of the participants who regularly read articles had adequate knowledge regarding epidemiology as compared to 23.3% of the participants who did not do any reading (p-value <0.001) [4]. In our study, previous experience had no significant (p > 0.05) effect on knowledge of epidemiology. According to a study done by Segal S et al., residents with prior experience of conducting research were more likely to participate in research projects during residency as compared to residents who did not have any prior experience of conduction research (p-value < 0.001) [8].

Inappropriate knowledge in our study was related to a low understanding of questions with regards to study design and statistical analysis. In order to conduct a good quality research, it is imperative to have a proper understanding of study designs and statistical analysis [18]. Research is fundamental for residency training but despite this fact, it has taken a back seat among residents. In a study conducted in Japan among physicians, it was concluded that training in clinical research is important for improving knowledge about epidemiology [19]. In Pakistan, research is in the initial stages and we are experiencing the same obstacles as experience by other third world countries. There is very limited research work due to poor research training [20]. 

## Conclusions

Most of the participants had an average knowledge of epidemiology, which is the basis of research. Knowledge of epidemiology is highly important for future research projects in Pakistan. There is a dire need to change the curriculum of medical schools and residency training programs in order to promote a research culture.
